# Oxygenator assisted dynamic microphysiological culture elucidates the impact of hypoxia on valvular interstitial cell calcification

**DOI:** 10.1186/s13036-024-00441-4

**Published:** 2024-08-23

**Authors:** Claudia Dittfeld, Florian Schmieder, Stephan Behrens, Anett Jannasch, Klaus Matschke, Frank Sonntag, Sems-Malte Tugtekin

**Affiliations:** 1 Department of Cardiac Surgery, Faculty of Medicine and University Hospital Carl Gustav Carus, Technische Universität Dresden, Heart Centre Dresden, Dresden, Germany; 2 Fraunhofer Institute for Material and Beam Technology IWS, Dresden, Germany

**Keywords:** Calcific aortic valve disease, Calcification, Human valvular interstitial cells, Hypoxia, Dynamic cell culture, Microphysiological system

## Abstract

**Introduction:**

Microphysiological systems (MPS) offer simulation of (patho)physiological parameters. Investigation includes items which lead to fibrosis and calcification in development and progress of calcific aortic valve disease, based e.g. on culturing of isolated valvular interstitial cells (VICs). Hypoxia regulated by hypoxia inducible factors impacts pathological differentiation in aortic valve (AV) disease. This is mimicked via an MPS implemented oxygenator in combination with calcification inducing medium supplementation.

**Methods:**

Human valvular interstitial cells were isolated and dynamically cultured in MPS at hypoxic, normoxic, arterial blood oxygen concentration and cell incubator condition. Expression profile of fibrosis and calcification markers was monitored and calcification was quantified in induction and control media with and without hypoxia and in comparison to statically cultured counterparts.

**Results:**

Hypoxic 24-hour culture of human VICs leads to HIF1α nuclear localization and induction of EGLN1, EGLN3 and LDHA mRNA expression but does not directly impact expression of fibrosis and calcification markers. Dependent on medium formulation, induction medium induces monolayer calcification and elevates RUNX2, ACTA2 and FN1 but reduces SOX9 mRNA expression in dynamic and static MPS culture. But combining hypoxic oxygen concentration leads to higher calcification potential of human VICs in calcification and standard medium formulation dynamically cultured for 96 h.

**Conclusion:**

In hypoxic oxygen concentration an increased human VIC calcification in 2D VIC culture in an oxygenator assisted MPS was detected. Oxygen regulation therefore can be combined with calcification induction media to monitor additional effects of pathological marker expression. Validation of oxygenator dependent VIC behavior envisions future advancement and transfer to long term aortic valve tissue culture MPS.

**Supplementary Information:**

The online version contains supplementary material available at 10.1186/s13036-024-00441-4.

## Introduction

Microphysiological or Organ-on-a-Chip systems are innovative in vitro platforms simulating in situ processes and complement in vivo model systems. Pathological transformations are mimicked and pharmacological testing is realized for the development of substance-based treatment strategies in cardiovascular research [1]. Also, investigation of calcific aortic valve disease (CAVD) in dynamic cell or tissue culture in MPS is intensified [2–4]. CAVD is the most frequent heart valve pathology and the third most common cardiovascular disease with increasing global incidence, prevalence and deaths [5, 6]. Since decades researchers investigate the pathological mechanisms leading to fibrosis and calcification of three-layered AV tissue taking the extreme biomechanics into account. But still the surgical AV replacement or transcatheter AV implantation are the only treatment options without the possibility for a medical intervention to stop disease progression [7, 8]. Cellular driven osteogenesis and resulting mineralization by enrichment of calcium hydroxyapatite in the tissue matrix became focus of AV research [9, 10]. Valvular interstitial cells (VIC) possess the potential to osseous differentiation and are assumed to be pathological mediators and players [11, 12]. In healthy tissue VICs maintain the extracellular matrix structure and functionality. In pathological tissue dystrophic calcification (via apoptotic processes) and neoosteogenesis resulting in leaflet stiffening are induced [13, 14]. Nevertheless, a potential impact of migrating cells such as mesenchymal stem cells is possible [15]. CAVD pathology is influenced by multiple factors starting from destruction of the cusp encompassing endothelial cell layer or endothelial to mesenchymal transition and ending with VIC related disorganization of ECM or immunological and biomechanical features [8, 16]. Enrichment of collagen and fibrotic ECM disorganization with resulting leaflet thickening can cause hypoxia in the AV tissue [17, 18]. But also, hypoxia can contribute to VIC myofibroblast differentiation leading to altered expression of α-smooth muscle actin (αSMA or ACTA2), fibronectin (FN1), collagen 1 and 3 (COL1A1, COL3A1) vice versa. Recently the impact of hypoxic oxygen concentration on human VIC calcification was investigated in conventional cell culture setups modulating the oxygen concentration or chemically activate HIF1α (hypoxia inducible factor 1 α) but also HIF2α [19]. HIF1α and HIF2α were shown to also be induced by phosphate rich calcification induction medium (OM). Both hypoxia and OM resulted in osteogenesis transcription factor RUNX2 (runt related transcription factor 2) induction but also in the induction of SOX9 (SRY-box transcription factor 9) that rather has been described to prevent AV calcification and regulates processes of chondrogenesis [19, 20]. As a result, calcification of hVIC cell culture was increased already after 24-hour incubation in hypoxic condition [19]. However, tissue oxygen concentration is of relevance for CAVD processes via HIF1α regulation [17]. Healthy cusp tissue does merely possess vessels near annulus structure in the thicker annealing leaflet [13, 21–23]. In contrast neovascularization is common during pathological disorganization of CAVD tissue [14, 24, 25] and has been proven also verifying the expression of HIF1α [26]. HIF1α induction in porcine AV tissues was shown for an oxygen concentration of 13% (arterial blood) after static AV tissue culture [27]. In elderly, porcine AV tissues hypoxia was related to pathological remodeling processes also in 13% oxygen [28]. Given the possibility to modulate the oxygen concentration in MPS, related pathological tissue transformation of CAVD can be investigated, because fibrotic tissue thickening is a common process and the undersupply with oxygen is assumed. Due to the AV tissue anatomy with a cusp tissue thickness of < 1 mm the application of bioreactors or smaller scaled tissue culture concepts is required. Recently, an MPS including a tissue incubation chamber was introduced by the authors, realizing the dynamic culture of porcine AV tissue segments to investigate CAVD pathology with approximation of physiological conditions [4]. Modulation of oxygen concentration during tissue culture is projected herein based on a 2D cell culture MPS, mimicking hypoxia via an oxygenator system implemented in the chip itself [29]. Aim of the study was to verify the impact of tuned hypoxic, normoxic and arterial blood oxygen concentration on mRNA expression of human VICs in comparison to cell culture incubator oxygen conditions using this oxygenator-chip in a modular setup. In addition, cell culture media to induce calcification in hVICs and effect of hypoxia on calcification induction was monitored in the MPS.

## Materials and methods

### VIC isolation and culture

Human VICs were isolated from human aortic valves explanted during surgical AV replacement (ethical votum EK429102015; *n* = 27, 67.5 ± 7.7 years old, 7 female and 20 male patients) as described previously [30]. Non-calcified tissue subsegments were used. In brief the endothelial cell layer was removed with a mixture of collagenase (Serva, Collagenase NB8 Broad Range 0.3 PZ U/ml) and dispase II (Sigma; 0.81 U/mg) and tissue was minced with scalpels. With a second collagenase digestion cells were released from matrix, suspension was filtered and collagenase was inactivated by addition of fetal calf serum (10%, FCS). VICs were plated on collagen type 1 (BD) coated culture plastic in Dulbecco’s Modified Eagle’s Medium (DMEM) supplemented with 10% FCS. Passages two to four were used for experimental setup.

### Experimental setup VIC MPS culture

VICs isolated from AV tissue of one individual were used per culture experiment. All culture plastics including MPS chips were collagen type 1 coated prior cell seeding. Equivalent hVIC numbers of 400 cells/mm^2^ for the 24-hour incubation and 200 cells/mm^2^ for the 96-hour incubation were seeded 12 h before the start of experiment for cell adherence.

**Fig. 1:**
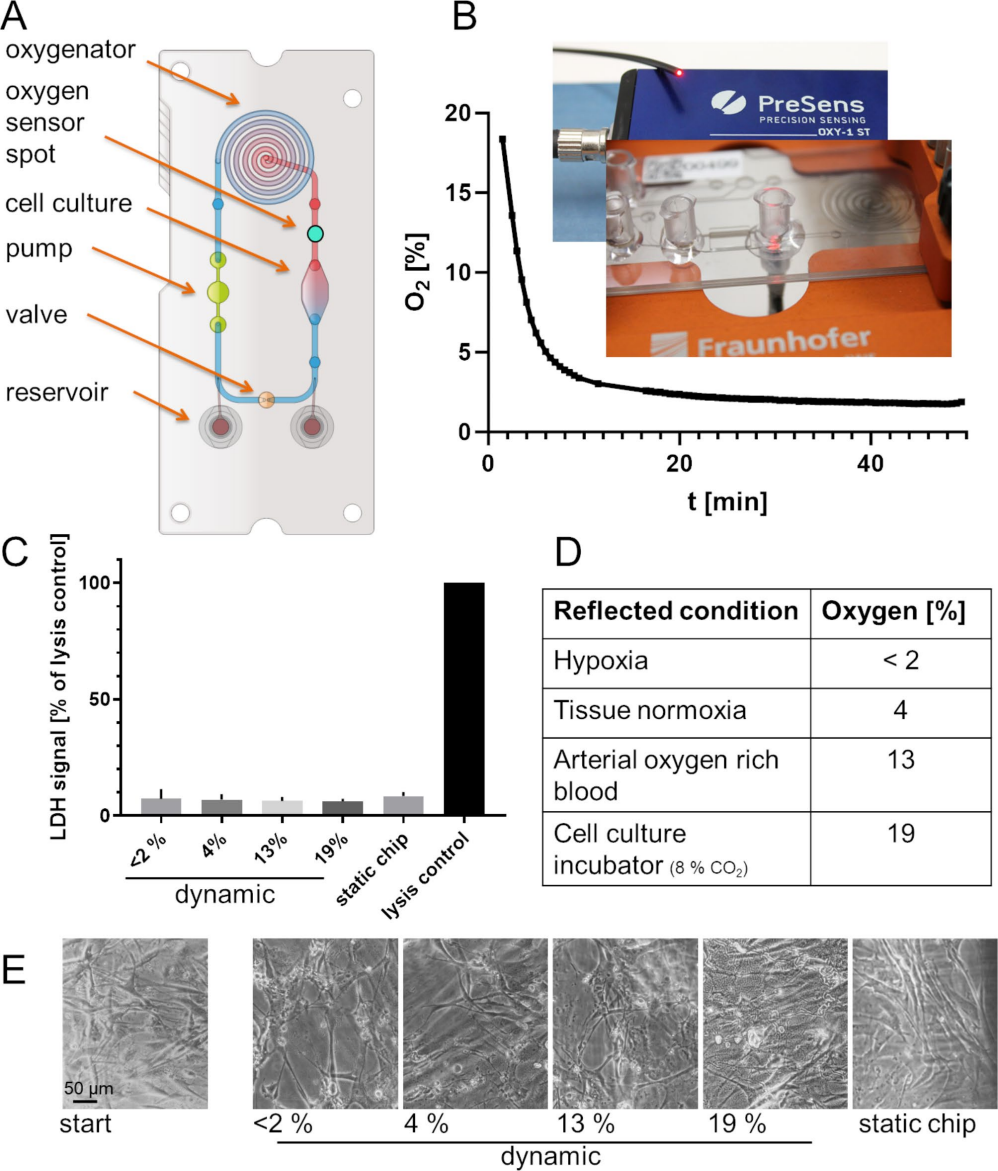
Oxygen regulation and monitoring in the oxygenator assisted MPS for hVIC culture. (**A**) multilayer-based microdevice, with a pneumatic pump system, an oxygenator and a cell culture chamber for dynamic culture, (**B**) Detection of oxygen concentration is realized via an oxygen sensor spot and by non-invasive continuous measurements with OXY-1 SMA Fiber Optic Oxygen Meter. Example of adjustment of hypoxic oxygen concentration within 30 min. (**C**) LDH-based cell viability measured by enzyme release in the cell culture supernatant. Death cell control signal was significantly higher than for all other cell culture conditions (not marked), that did not differ among each other (one-way ANOVA; * *p* < 0.05) (**D**) Adjusted oxygen concentrations for 24-hour incubation reflecting (patho)physiological conditions. (**E**) Exemplary hVIC culture after 24-hour culture in the respective conditions

A MPS consisting of control unit, device holder multilayer-based microdevice (chip, Fig. 1A) with a pneumatic pump system and an oxygenator was applied. Luer connectors are used for cell seeding and medium supply (Fig. 1A and B). A pulsatile flow (2.7 µl/s; average shear forces of 0.04 dyn/cm^2^, 40 bpm) was generated via deflection of the 200-µm thick, flexible, gas-permeable pump membrane that was laminated in-between stacks of laser-cut polycarbonate foils [29, 31]. Regulated medium oxygen concentration was determined via an oxygen sensor spot (SP-PSt7; PreSens, Regensburg, Germany) by non-invasive continuous measurements with OXY-1 SMA Fiber Optic Oxygen Meter (Presens, Fig. 1B). A cell culture medium reservoir was connected via Luer connection also serving as a bubble trap.

#### Experimental setup 1

Evaluating the impact of oxygen concentration on VIC marker expression, < 2%, 4%, 13% and 19% oxygen were regulated in the four dynamically cultured MPS chips for 24 h. This corresponds to < 20.1 µMol, 40.2 µMol, 130.6 µMol and 190.8 µMol O_2_ (Oxygen Unit Calculator, Excel, Presens). A static chip control was implemented, not connected to the fluidic system and conventional 96-well plate static controls were performed. Oxygen concentration of conventional cell incubator condition with humidified atmosphere and bicarbonate buffering was calculated 19%. Setup was repeated with three (immunofluorescence analysis, *n* = 3) to eight (expression analysis, *n* = 8) human VIC preparations.

#### Experimental setup 2

To verify the impact of dynamic culture on calcification potential of culture media ADGM (50 µM **A**scorbic acid phosphate, 100 nM **D**examethasone, 10 mM β-**G**lycerophosphate) and PM (2 mM sodium dihydrogen phosphate, pH 7.4, and 0.3 mM ascorbic acid) were used in dynamic MPS and static culture for 96 h in comparison to conventional DMEM all containing 10% FCS.

#### Experimental setup 3

To investigate the combined effect of reduced oxygen concentration on in vitro calcification potential solely ADGM was selected and dynamically incubated in < 2% and 19% oxygen for 96 h in comparison to DMEM counterparts using one individual human VIC preparation per experiment. Equivalently static controls were investigated in the 96-well plate format.

### LDH cell viability assay

CytoTox-ONE™ Homogeneous Membrane Integrity Assay (Promega, G7890) was applied according to manufacturer’s instructions. The release of lactate dehydrogenase (LDH) from cells with a damaged membrane is quantified in comparison to a death cell control by adding 1:50 Lysis Solution (9% [weight/volume] Triton^®^ X-100 in water).

### Immunofluorescence staining of VICs in MPS-chips

To monitor HIF1α nuclear localization hVICs were seeded and treated according to experimental setup 1. Cell viability was proven by LDH membrane integrity assay from removed cell culture medium. Cells were washed with 1 ml PBS (4 °C) and fixation was performed using methanol (-20 °C) incubation for ten minutes. After removal of methanol chips with fixed cells were air dried and stored at -20 °C until immunofluorescence staining. Therefore, chips were defrosted and air dried and luer lock connectors were removed. Cell culture chamber and fixed cells were washed three times with 150 µl PBS prior incubation with 150 µl 0.2% Triton-X100 (3mM) in PBS. After three times PBS washing step, blocking was performed in 1% bovine serum albumin (BSA) in PBS for 30 min. HIF1α polyclonal antibody (ab16066, abcam) was diluted to 5 µg/ml in BSA-PBS, 80 µl of the solution were applied to the chip cell chamber and incubated for one hour at room temperature. Normal rabbit IgG isotype control antibody (Cell Signalling Technology, #2729) was used as an isotype control in a static condition well diluted to the respective antibody concentration. After three times washing with PBS, Alexa Fluor^®^ 488 labelled secondary antibody (abcam, ab150073) was diluted 1:500 in BSA-PBS and 80 µl of the solution were incubated for one hour at room temperature. Cell nuclei were DAPI (4′,6-Diamidin-2-phenylindol, Thermo Fisher, D3571) stained using a 1.3 µM dilution in PBS for five minutes. After additional washing embedding was performed after removal of upper chip polycarbonate layer using fluorescent mounting medium and a cover glass. Rate of positive nuclei was determined by manually counting the DAPI positive nuclei of one field of view, change the filter set and count the HIF1α-positive nuclei and proving DAPI positivity repeatedly using fluorescence microscopy. Chip cell culture chamber was analyzed according a raster system. Number of HIF1α-positive cell nuclei was related to total number of cell nuclei at each condition.

### Quantification of calcium concentration

After respective culture period cell medium was removed, the chips and dishes were washed twice with calcium free DPBS and 360 µl 0.1 M nitric acid were added and incubated overnight. Calcium ion concentration was quantified using a Spektro quant Calcium-Test Kit (Merck KGaA, 1.00049.0001), according to the manufacturer’s instructions and after respective dilution. The calcium content was related to total protein by removing the nitric acid solution, washing the wells twice with DPBS, adding 150 µl of a 0.05 M NaOH/0.1% SDS solution per chip. Protein concentration was determined after incubation overnight at RT in a 96-well format using a Pierce BCA Protein Assay Kit (Thermo Fisher Scientific) according to the manufacturer’s instructions. For better comparability, the results were calculated in units of mol Ca^2+^/(kg protein).

### Quantitative RT-PCR

RNA isolation from hVICs chip and 96-well-plate culture was performed by addition of 3 × 100 µL of peqGold RNAPure (peqlab, 30-1010) and application of the InviTrap Spin Universal RNA Mini Kit (Stratec molecular, 1060100300) according to the manufacturer’s instructions. RNA concentration was determined using Qubit Fluorometer after probes were diluted in 200 µl TAE-buffer containing 1 µl QuantiFluor^®^RNA Dye.

For cDNA synthesis, mRNA template was harmonized according to concentration and transcribed into cDNA using Maxima First Strand cDNA Synthesis Kit for RT-qPCR (Thermo Fisher Scientific, #K1672) according to manufacturer’s instructions (Oligo[dT]18 and random hexamer primers). Reaction was performed as follows: 25 °C for 10 min, primer annealing and elongation at 50 °C for 15 min. Reaction was terminated for 5 min at 85 °C. Complementary DNA was amplified using MESA Green qPCR Mastermix Plus for SYBR^®^Assay No ROX (Eurogentec, RT-SY2X-03 + NRWOU) according to manufacturer’s instructions. 7.5 µl reaction master mix (2x) were used for a total volume of 15 µl containing template and 250 nM of forward and reverse primer, respectively. The reaction was performed in a Rotor-Gene-Q (Qiagen) as follows: denaturation at 95 °C for 5 min, 40 cycles of denaturation at 95 °C for 15 s and one minute primer annealing and elongation at 60 °C. Each sample was analyzed in duplicates. Reaction was verified by melting curve control. Different dilution steps up to 1:1000 were measured for generating calibration curves and to calculate reaction efficiencies using Rotor-Gene Q software. Delta/delta-Ct method was used implementing reference gene expression of GAPDH (Glyceraldehyde 3-phosphate dehydrogenase), PPIA (Peptidyl-propyl-isomerase A), HPRT (Hypoxanthine-guanine-phosphoribosyltransferase) and 18 S (RNA, 18 S ribosomal 5) to calculate expression of marker genes HIF1α (hypoxia inducible factor 1 α), HIF2α (hypoxia inducible factor 2 α), EGLN1-3 (egl nine homolog 1–3 or PHD2, 1, 3,prolyl hydroxylase enzymes), LDHA (Lactatdehydrogenase A), ACTA2 (α-smooth muscle actin [αSMA]), FN1 (fibronectin1), COL1A1 (Collagen 1), COL3A1 (Collagen 3), RUNX2 (Runt-related transcription factor 2) and SOX9 (SRY-box transcription factor 9) in relation to respective control condition and presented as x-fold change. Primer sequences are given in Table 1.

**Table 1:** Primer sequences used for qRT-PCR

Gene	Primer	Sequence (5’-3’)
**Reference genes**		
18s	forward	TCCGACCATAAACGATGCCGAC
	reverse	GGTGAGGTTTCCCGTGTTGAGT
GAPDH	forward	CAAGGGCATCCTGGGCTA
	reverse	CCACCACCCTGTTGCTGTAG
HPRT	forward	TGGACAGGACTGAACGTCTT
	reverse	GAGCACACAGAGGGCTACAA
PPIA	forward	CATACGGGTCCTGGCATCTT
	reverse	GGTGATCTTCTTGCTGGTCTTG
**Genes of interest**		
ACTA2	forward	TCAATGTCCCAGCCATGTAT
	reverse	CAGCACGATGCCAGTTGT
Col1a1	forward	GCTCCTGGTATTGCTGGTG
	reverse	CCAGGTTCACCGCTGTTAC
Col3a1	forward	TAGGTCCATCTGGTCCTGCT
	reverse	ATCGAAGCCTCTGTGTCCTT
EGLN1	forward	TGAGCAGCATGGACGACCTGAT
	reverse	CGTACATAACCCGTTCCATTGCC
EGLN2	forward	CTGTCTGGTATTTTGATGCCAAGG
	reverse	CGGCTGTGATACAGGTACTTGG
EGLN3	forward	GAACAGGTTATGTTCGCCACGTG
	reverse	CCCTCTGGAAATATCCGCAGGA
FN1	forward	CGGTGGCTGTCAGTCAAAG
	reverse	AAACCTCGGCTTCCTCCATAA
Hif1a	forward	CATAAAGTCTGCAACATGGAAGGT
	reverse	ATTTGATGGGTGAGGAATGGGTT
Hif2a	forward	AGTTCACCTACTGTGATGACAGA
	reverse	GACCCTTGGTGCACAAGTTC
LDHA	forward	AGCCCGATTCCGTTACCT
	reverse	CACCAGCAACATTCATTCCA
Runx2	forward	GGTACCAGATGGGACTGTGG
	reverse	GGTGAAACTCTTGCCTCGTC
Sox9	forward	GAACGCACATCAAGACGGAG
	reverse	AGTTCTGGTGGTCGGTGTAG

Statistics: Evaluation of mRNA expression according to oxygen concentration and dynamic vs. static culture was performed with *n* = 8. mRNA expression experiments in PM or ADGM at dynamic vs. static culture and HIF1α nuclear localization were performed at least in triplicates. Calcification in ADGM and PM in dynamic vs. static culture was quantified in quadruplicates and in dependence of oxygen concentration with *n* = 6. Resulting data were stated as mean ± standard deviation. Hypothesis testing for the assessment of statistical significance was computed by variance analyses with one- or two-way ANOVA, Kruskal-Wallis or Friedman test´s according to normality pretesting of respective datasets. Post-hoc analyses with Tukey multiple comparison testing using the software PRISM (Graphpad Software, Inc., USA). Null hypotheses (H0) were rejected if *p* < 0.05 (See Table 1).

## Results

### Oxygenator assisted hypoxic VIC MPS-culture

Human VICs were seeded into chip cell culture chamber (Fig. 1A and B) 12 h prior experiment start to achieve cell adherence. LDH release to culture medium as a marker for cell viability was verified prior experiment start 12 h after seeding (not shown) and after experiment termination to elucidate an impact of the different oxygen concentrations (Fig. 1C; death cell control was set as 100% LDH release). Since the chip deflection membrane is gas permeable and air-pressure driven, nitrogen was both conducted to the pneumatic pump and the oxygenator to achieve oxygen concentrations below 8%. To simulate hypoxia, oxygen concentrations below 2% were applied in the dynamic pulsatile MPS (Fig. 1B and D). Tissue normoxia was reflected by adjustment of 4% and arterial blood oxygen concentration by 13% oxygen in the cell culture media. Conventional cell culture in DMEM demands a CO_2_ buffering resulting in an incubator oxygen concentration of approximately 19% oxygen (Fig. 1D) [32]. To directly compare dynamic vs. static cell culture condition, 19% oxygen was adjusted in the dynamic MPS control. Target media oxygen concentration of < 2% was monitored non-invasively, adjusted in 20 to 120 min and preserved for 24–96 h in experiments culturing VICs (Fig. 1B). The mean final oxygen concentrations after 24 h incubations were 1.4 ± 0.4%, 4.6 ± 0.6%, 13.0 ± 2.9%, 17.1 ± 2.1%, respectively and for the static chip control not regulated via the oxygenator 17.1 ± 2.1% (not shown). The mean final oxygen concentration for the 96 h incubation was 0.9 ± 0.2% and 19.5 ± 1.6%, respectively and for the static chip control 18.1 ± 1.2% (data not shown). Valvular interstitial cell culture was verified microscopically and revealed no apparent changes (Fig. 1E).

### Hypoxia related VIC calcification and marker expression in MPS-culture

**Fig. 2:**
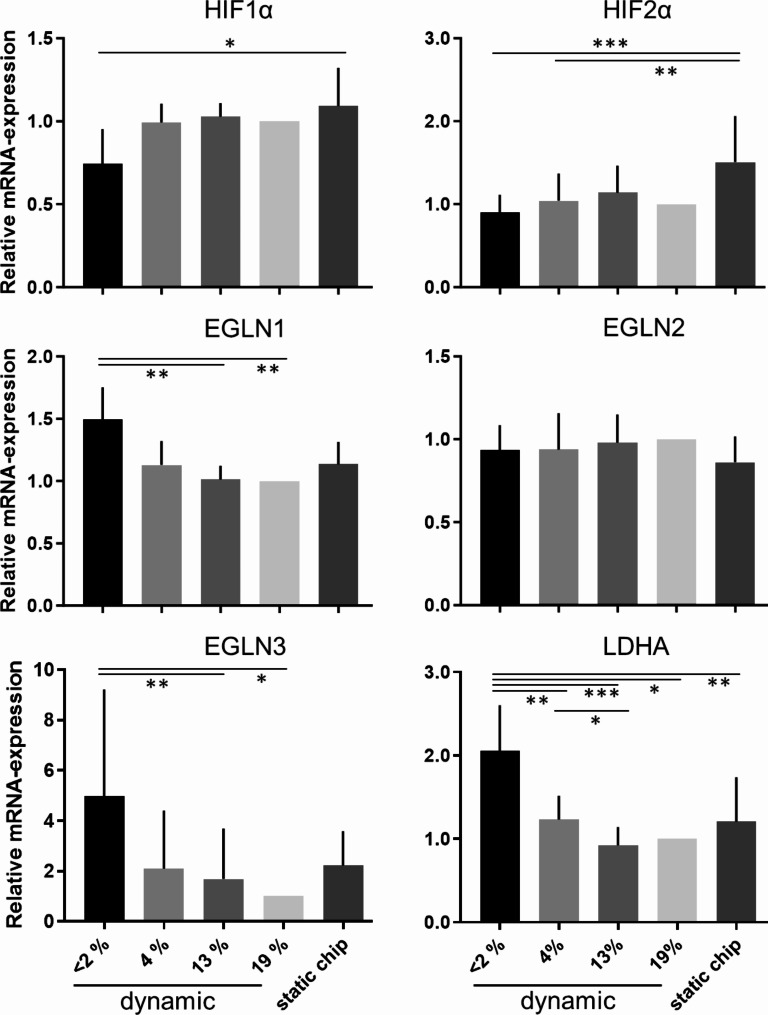
Relative mRNA expression of genes regulated by hypoxia in hVICs cultured for 24 h in dynamic MPS culture with different oxygen concentrations in comparison to static control and related to dynamic MPS culture at 19% oxygen. Hypoxia inducible factors HIF1α and HIF2α and its regulators EGLN1,2,3 (prolyl hydroxylases) and the HIF effector LDHA were quantified. (one-way ANOVA and Tukey-test or Friedmann and Dunn`s-test, respectively; *p < 0.05; **p < 0.01; ***p < 0.005; ****p < 0.0001)

mRNA expression of hypoxia related genes was verified in dynamic hVIC culture in MPS and in static chip and 96-well-plate controls after 24 h. Dynamic MPS culture was performed at < 2, 4, 13 and 19% oxygen vs. static chip format at conventional cell culture condition. Respective marker mRNA expression level was related to dynamic 19% condition (Fig. 2). Hypoxia inducible factor mRNA expression did not differ between the dynamic setups at hypoxic, nomoxic or arterial blood oxygen levels compared to conventional cell culture values- HIF1α mRNA was significantly lower (0.7 ± 0.2) in dynamic hypoxic condition compared to static chip control (1.1 ± 0.2). Also, the HIF2α mRNA was with 1.5 ± 0.6 significantly higher expressed in static chip control comparing hypoxic dynamic condition (Fig. 2). HIF prolyl hydroxylases cause hydroxylation and subsequent HIF ubiquitylation and destruction and are also regulated by oxygen concentration [33]. In contrast to HIF, these hypoxia related markers, EGLN1 and EGLN 3, are with 1.5 ± 0.3 and 4.7 ± 4.0, respectively, significantly elevated comparing the dynamically cultured counterparts at 19% oxygen set as control. EGLN2 mRNA expression did not depend on medium oxygen level. LDHA, a HIF1α regulated effector, is up to two times higher expressed (2.0 ± 0.6) in the hypoxic condition compared to all other conditions evaluated. Already at the 4% condition there is a significant higher LDHA mRNA expression compared to 13% dynamically incubated hVICs. Expression of myofibroplast and ECM markers ACTA2, FN1, COL1A1 and COL3A1 but also expression of markers RUNX2 and SOX9, related to osteoblast transformation is not regulated according to oxygen concentration in hVIC culture (Supplementary Fig. 1).

**Fig. 3:**
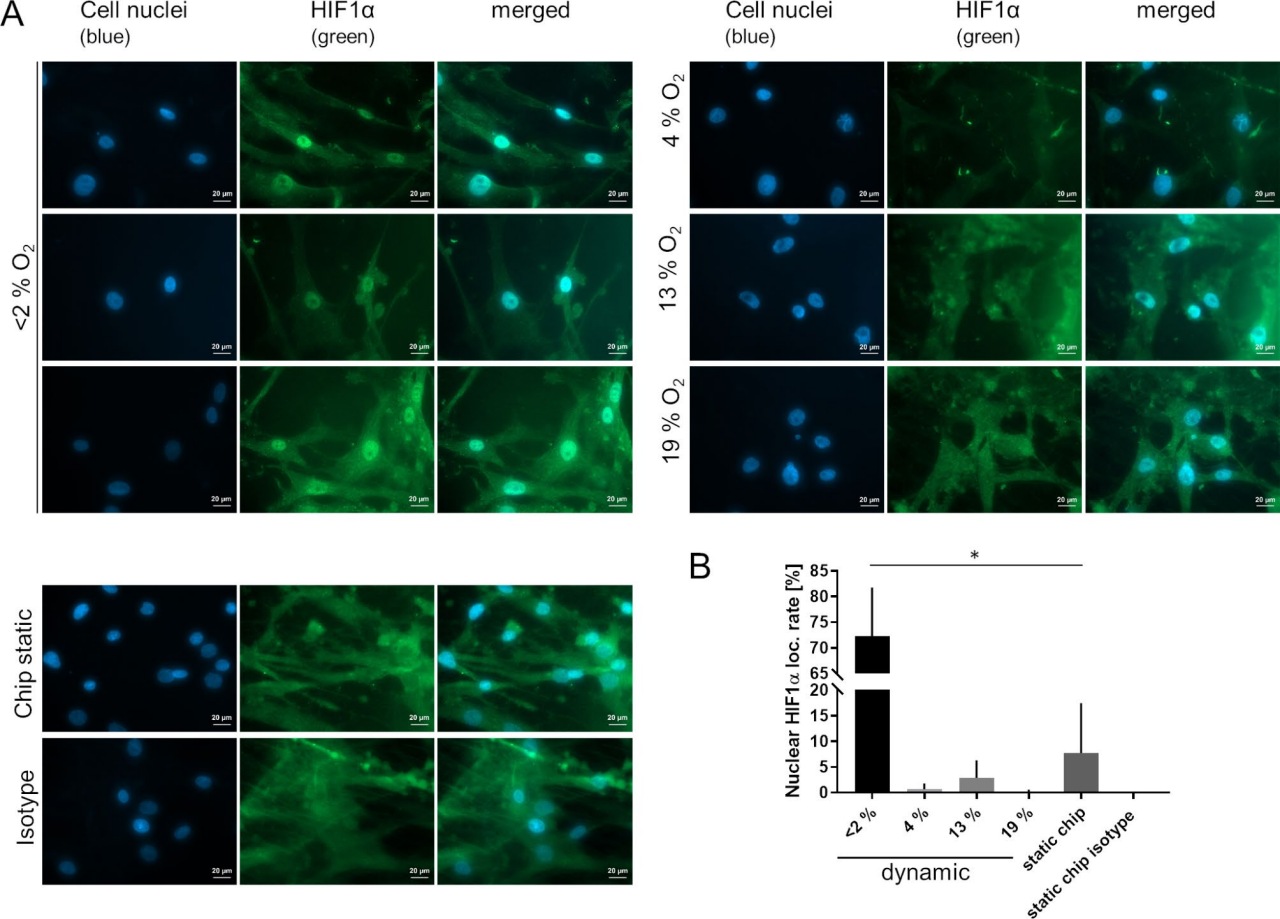
HIF1α nuclear localization in hVICs cultured in dynamic MPS hypoxic condition visualized via immunofluorescene in comparison to isotype control. (**A**) HIF1α nuclear localization was merely detected in hVICs cultured under hypoxia. No or very low rates of nuclei were HIF1α positive at oxygen ≥ 4%. (**B**) Quantification of HIF1α positive nuclei rate. (Friedmann and Dunn`s-test, *p < 0.05)

Since hypoxic marker HIF1α mRNA expression was not elevated after 24 h in hypoxic dynamic culture protein localization was investigated via immunofluorescence staining of fixed hVICs. Nuclear localization was mainly visible in < 2% oxygen culture (Fig. 3A). Rate of HIF1α positive cell nuclei was determined and revealed a significant higher rate of HIF1α positive nuclei (72.3 ± 9.5%) in hypoxic condition compared to 19% oxygen counterpart (0.2 ± 0.4%; Fig. 3B). Therefore, activation of HIF1α transcription factor is assumed.

### Media induced VIC calcification and marker expression in MPS-culture

**Fig. 4:**
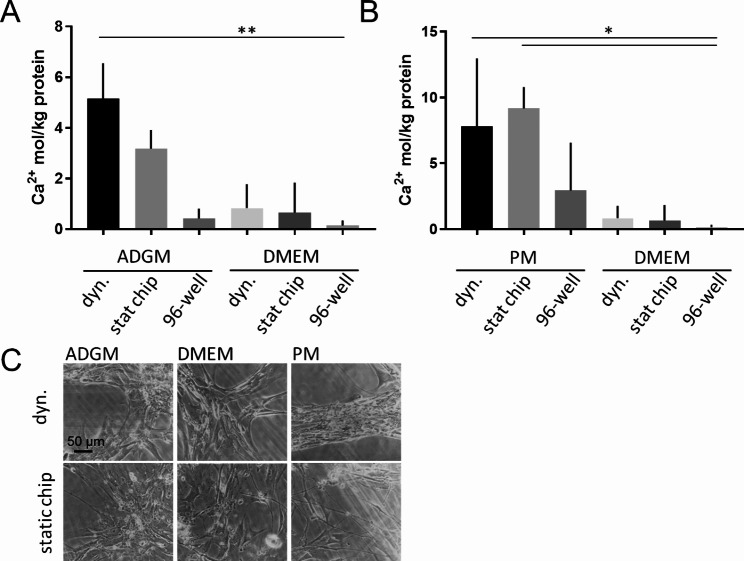
Induction of calcification of 2D hVIC cultures in dynamic MPS culture in comparison to static counterparts (chip static control and 96-well format). (**A**) Calcium concentration was quantified and related to protein concentration after 96-hour incubation in ADGM and (**B**) in PM and control medium (Friedmann and Dunn`s-test; *p < 0.05; **p < 0.01). (**C**) Cell culture was evaluated microscopically

Assays to investigate VIC calcification potential in vitro apply special media formulations increasing availability of phosphate ions or using ascorbic acid and dexamethasone to induce osseous differentiation [30, 34]. ADGM including ascorbic acid and dexamethasone or PM without dexamethasone were used in comparison to conventional DMEM full medium herein to investigate calcification potential of hVICs in dynamic vs. static culture for 96 h (Fig. 4). Calcium content of cell culture layer cultured dynamically in ADGM differed with 5.1 ± 1.4 mol Ca^2+^/kg protein significantly from static conventional DMEM condition (0.1 ± 0.2 mol Ca^2+^/kg protein). An insignificant increase was observed in the static ADGM chip culture. Comparing the PM condition significant induction of calcification was observed for both dynamic (7.8 ± 5.2 mol Ca^2+^/kg protein) and static chip (9.2 ± 1.6 mol Ca^2+^/kg protein) compared to static DMEM control was detected. Nevertheless, induction of calcification by ADGM or PM in dynamic vs. static culture did not differ.

**Fig. 5:**
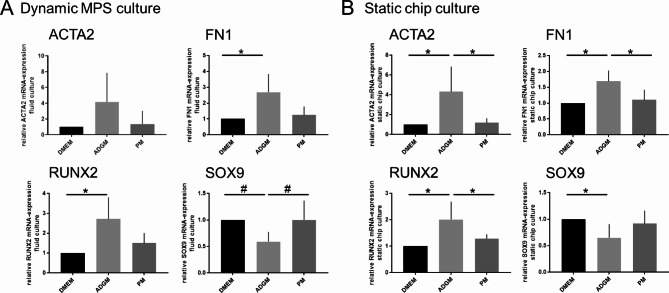
Relative marker mRNA expression in dynamic vs. static MPS culture of hVICs in calcification induction media ADGM and PM. (**A**) Changes of expression in dynamic MPS culture comparing DMEM, ADGM and PM for marker genes ACTA2, FN1, RUNX2 and SOX9, (**B**) respective mRNA expression set in static MPS chip culture. (one-way ANOVA and Tukey-test or Friedmann and Dunn`s-test, respectively; *p < 0.05; **p < 0.01; # *p* < 0.1)

**Fig. 6:**
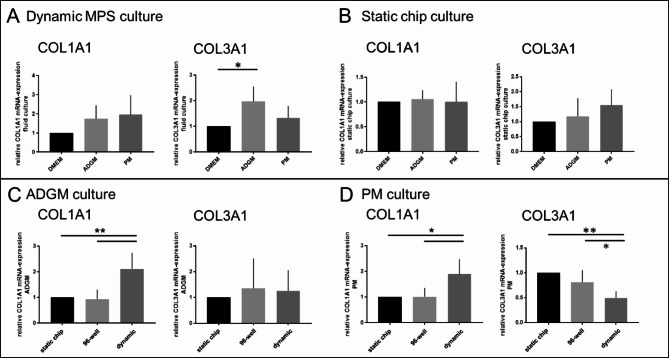
Relative marker expression in dynamic vs. static MPS culture of hVICs in calcification induction media ADGM and PM. (**A** and (**B**) COL1A1 and COL3A1 expression is not only evaluated comparing different media in dynamic and static culture but also; (**C** and **D**) comparing dynamic and static culture implementing the 96-well static setup in addition and according to ADGM and PM. (one-way ANOVA and Tukey-test or Friedmann and Dunn`s-test, respectively; *p < 0.05; **p < 0.01; # *p* < 0.1)

Marker expression was determined at mRNA level. Markers ACTA2 and FN1 are elevated to 4.2 ± 3.7 (dynamic) or 3.1 ± 1.1 (static) and 2.7 ± 1.2 (dynamic) or 1.6 ± 0.3 (static) only in the ADGMedium but independent from dynamic or static culture (Fig. 5). As observed in the first experimental setup investigating the impact of oxygen concentration on culture, also here there is a significant higher mRNA expression of ACTA2 and FN1 in conventional 96-well plate format comparing DMEM condition (not shown). Also, RUNX2 is increased in ADGM condition to 2.7 ± 1.1 (dynamic) and 2.0 ± 1.1 (static) and chondrogenesis marker SOX9 is reduced in both dynamic and static condition by trend or significantly to 0.6 ± 0.2 and 0.7 ± 0.1 after incubation in ADGM (Fig. 5). COL3A1 mRNA expression was significantly higher (2.0 ± 0.6) in dynamic ADGM culture compared to dynamic DMEM incubation (Fig. 6A). A reduction of COL3A1 to 0.5 ± 0.1 was observed comparing the PM static conditions with the dynamic incubation. Collagen 1 was the only marker that was upregulated only in dynamic culture for both induction media ADGM and PM to values of 2.1 ± 0.6 and 1.9 ± 0.6 respectively but not in static culture (Fig. 6C and D).

### Impact of hypoxic oxygen concentration on ADGM calcification induction

**Fig. 7:**
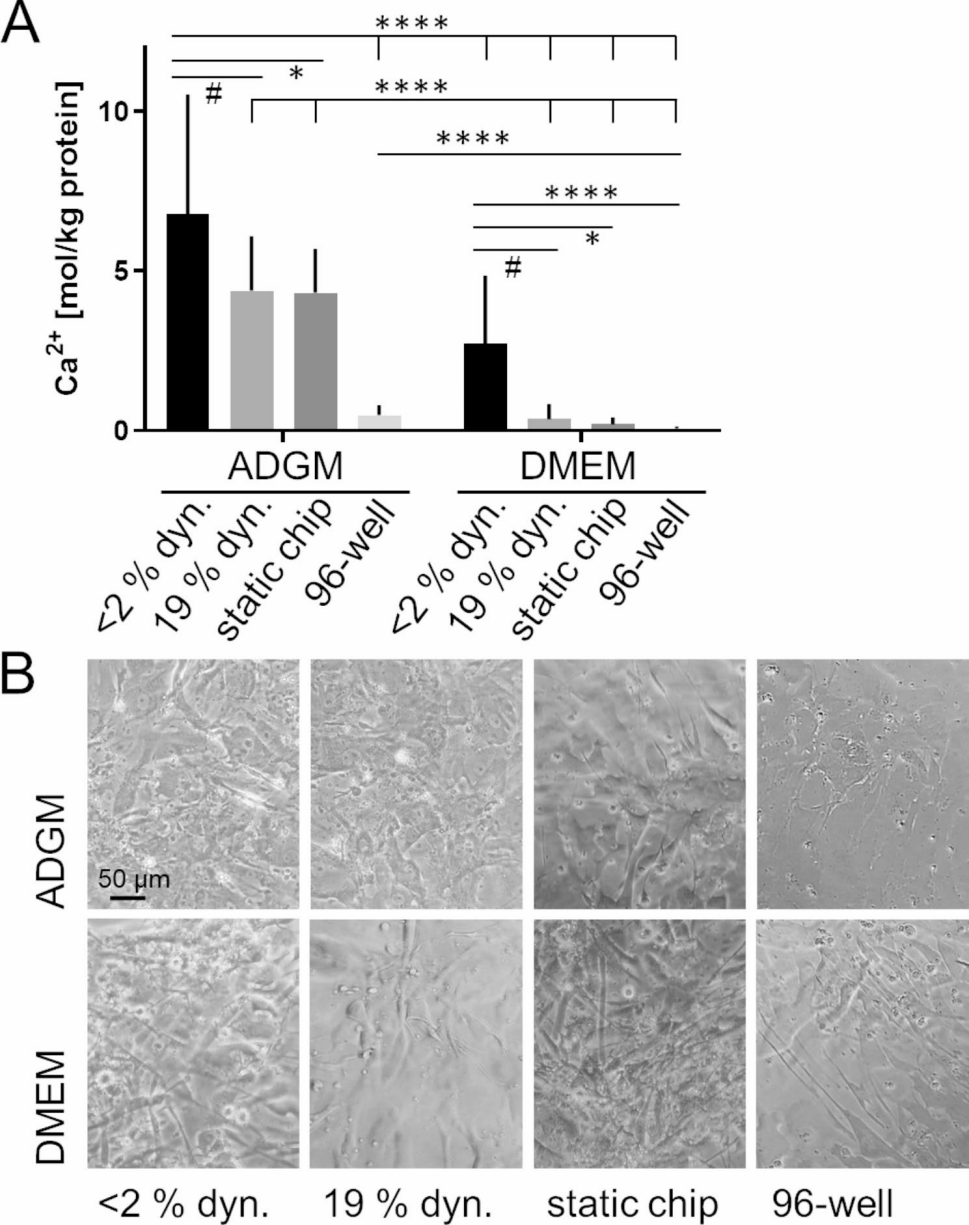
Induction of calcification of 2D hVIC cultures dependent on hypoxic and cell incubator oxygen concentration (19%) in dynamic MPS culture in comparison to static counterparts (chip static control and 96-well format). (**A**) Calcium concentration was quantified and related to protein concentration after 96-hour incubation in ADGM and control medium (two-way ANOVA and Tukey-test; *p < 0.05; ****p < 0.0001; # *p* < 0.1). (**B**) Cell culture was evaluated microscopically

Combining both, regulation of oxygen concentration and calcification induction, MPS was used to regulate hypoxic vs. incubator oxygen concentration in dynamic and static chip culture. ADGM was used as induction medium due to the regulation of mRNA expression of related markers. Significant increase of calcium induction was detected in the dynamic ADGM culture at < 2% oxygen (6.7 ± 3.8 mol Ca^2+^/kg protein) by trend differing from the dynamic ADGM culture at 19% oxygen (4.4 ± 1.7 mol Ca^2+^/kg protein) and significantly differing from static chip and 96-well format culture (4.3 ± 1.4 mol Ca^2+^/kg protein and 0.5 ± 0.3 mol Ca^2+^/kg protein, respectively) and all equivalent DMEM culture conditions (Fig. 7A; microscopical evaluation shown in Fig. 7B). Independent from biochemical induction in conventional DMEM also by trend a higher calcification concentration in hypoxic dynamic incubation (2.8 ± 2.1 mol Ca^2+^/kg protein) is observed compared to dynamic incubator oxygen condition (0.4 ± 0.5 mol Ca^2+^/kg protein) and significantly higher compared to both static incubation setups (0.2 ± 0.2 mol Ca^2+^/kg protein and 0.1 ± 0.1 mol Ca^2+^/kg protein, respectively). Since 19% conditions do not differ according to dynamic or static and comparing static 96-well format in neither the ADGM induction nor in the DMEM experiments an impact of hypoxic oxygen concentration is assumed.

## Discussion

Hallmark of early state CAVD pathology is tissue fibrosis leading to cusp thickening > 1 mm [35]. Since there are only few microvessels in the AV cusp tissue near annulus, the oxygen diffusion from oxygen rich arterial blood is dependent on biomechanics and can be reduced in the course of fibrotic ECM enrichment [17, 21, 36]. Low oxygen concentration is sensed by active cells. In AV leaflet tissue hypoxia can impact pathological differentiation in disease progress of VICs that are responsible for ECM maintenance in healthy tissue [17, 26, 31, 33]. Biomechanical alterations e.g. due to bicuspidal AV but also gender dependent fibrotic processes result in pathophysiological preconditioning [35]. Low oxygen availability is mainly transferred to a cellular reaction by regulation of hypoxia inducible factors [33, 37]. HIF1α is expressed ubiquitously in all cells and under normoxia, HIF1α is constantly synthesized and degraded, while under hypoxia, it is not degraded, stabilized and dimerizes with the β-subunit [38, 39]. Resulting heterodimer translocates into the nucleus, and together with coactivator molecules it binds to hypoxia response elements in HIF1 target genes and its transcription is activated [33, 38].

Nuclear localization of HIF1α in hVICs was demonstrated and proven in the oxygenator MPS at an oxygen concentration of < 2% herein, although an increase of HIF1α or HIF2α mRNA expression could not be verified. In contrast, oxygen dependent prolyl hydroxylases EGLN1 and EGLN 3, regulating HIF1α and HIF2α, respectively, are significantly elevated in the hypoxic, but not tissue normoxic hVIC MPS culture compared to arterial blood and cell culture incubator situation. EGLN1 regulation in hypoxic condition and induction of expression [40] has therefore been confirmed for hVICs. However, EGLN1 uses O_2_ as a substrate and thus Hif1 hydroxylation by EGLN1 and proteasomal degradation is dependent on oxygen availability [41]. EGLN2 mRNA expression is not affected by reduced oxygen concentration but lactate dehydrogenase (LDHA) is significantly induced at hypoxic oxygen concentration. LDHA converts pyruvate to lactate and is an effector gene of HIF1α metabolic regulation due to the transition from oxidative to glycolytic metabolism [37]. Expression analysis was performed after an incubation time of 24 h in the oxygenator MPS. Since HIF regulation is time dependent, experimental settings and opposing regulatory mechanism and induction of degrading activity of prolyl hydroxylases can impact HIF mRNA profile [33, 39]. In contrary culturing iPSC-derived cardiomyocytes (iPSC-CMs) for 48 h in the same oxygenator MPS in hypoxia resulted in a significant increase of HIF1α and EGLN2 expression [29]. Limited number of hVICs cultured in chip setup allows western blot analysis only if multiple chip cultures and therewith multiple individual hVIC preparations are pooled. This is envisioned to demonstrate not only induction of HIF1α and HIF2α by hypoxic oxygen concentration but also RUNX2 and SOX9 on protein level as performed recently by Csiki et al. [19]. RUNX2 has been identified as the master regulator switch in osteoblast differentiation and calcification [42] whereas SOX9 has been shown to mediate processes of chondrogenesis and prevents CAVD [20, 43, 44]. Hypoxia in oxygenator MPS did not induce changes in pathological marker expression in hVICs (independent hVIC isolations from eight aortic valves) neither analyzing expression of ECM genes that could reflect a fibrotic induction nor for RUNX2 or SOX9 regulation after 24 h. This is in contrast to other studies e.g. investigating vascular smooth muscle cells [45]. Contrary and as expected, RUNX2 mRNA expression is induced but SOX9 is decreased in ADGM initiating in vitro calcification of hVICs reflecting osteogenesis [30, 34]. Nevertheless, hVICs isolated from diseased tissues can be prone to calcification induction and related regulation. The heterogeneity of the hVIC preparations can also lead to early calcification induction in the cell culture model that is, as all VIC culture models, artificial. In contrast to the results of Csiki and colleagues RUNX2 or SOX9 induction was not observed in PM, reflecting the dystrophic process [19, 34]. Medium composition differs in these studies. According to previous results in our group and the data published by Goto et al., herein in both media compositions ascorbic acid was used, although an inhibiting impact on HIF transcription is possible due to the high affinity of HIF transcriptional regulator FIH (factor inhibiting HIF) to the substance [33, 37, 46]. Nevertheless, ascorbic acid concentration is very low and it is assumed that regulatory effect is marginal at least at very low oxygen concentration as shown for most of cell lines investigated [46]. Also, herein ADGM but not PM induced HIF1α mRNA expression significantly (data not shown) as detected for the ascorbic free induction medium in the study Csiki et al. [19]. Also, in vascular smooth muscle cells high levels of inorganic phosphate have been described to induce HIF. Calcification of the cells incubated in high phosphate media under hypoxic conditions increased the calcification process [45, 47, 48]. In general, beside hypoxia, HIF expression is also regulated under normoxia e.g. via growth factors, hormones, coagulation factors, cytokines, flow and inorganic phosphate [17, 39].

Static culture controls were applied to monitor the impact of chip system and medium flow at very low shear forces and flow rates. This merely guarantees oxygen concentration adjustment and does not reflect a physiological flow of an AV. However, low flow rates should effectively maintain oxygen concentration in cell culture chamber and balance potential oxygen VIC consumption effects. Nevertheless, future MPS design will include a second oxygen sensor spot to monitor oxygen concentration after cell culture chamber and therefore cells oxygen consumption. In addition very low flow rates were applied, since in cusps situated VICs are hardly exposed to flow. This is only possible if endothelial cusp layer is destroyed in CAVD processes. The static chip control at conventional cell culture conditions was evaluated to at least monitor impact of low flow on expression at ca. 19% oxygen concentration. Nevertheless, and although there was no oxygen dependent regulation in the dynamic vs. static chip culture, myofibroblast and ECM marker expression was higher for ACTA2 (α-smooth muscle actin), FN1 (fibronectin) and COL3A1 (collagen 3) and also HIF2α was elevated in the conventional 96-well cell culture setup (data not shown). Classical 2D cell culture concepts e.g. using high media supernatants are sources of artificial oxygen regulation [32] and have to be considered in detail. Conventional cell incubator situation with CO_2_ adjustment results in reduced atmospheric oxygen concentration, that still is artificial compared to in situ tissue [32]. Both induction media, ADGM and PM, induced calcification. This was independent from static or dynamic condition for hVICs in MPS culture. Quantification of calcium was used as the only calcification endpoint herein due to the complexity of the MPS and the previously published verification by histological staining (alizarinred and van-Kossa staining) and the chemical proof by FTIR-spectroscopy after induction by ADGM [30]. Highest calcification potential in ADGM was achieved in this setup in dynamic culture for 96 h at hypoxic condition, by trend differing from the cell incubator oxygen concentration also in dynamic culture. But even using DMEM, the impact of hypoxia on hVIC calcification in the oxygenator MPS was proven without elevated levels of inorganic phosphate. This makes the system most reliable for advancement of AV tissue culture application and studies to mimic in situ calcification process even without the need for chemical induction media.

Induction of hypoxia regulation and relevance of oxygen concentration for calcification process was validated for the culture of hVICs in the MPS. Limitations are on this cellular level the number and the diseased origin of the hVICs investigated. In addition, the experimental setup such as time point to prove HIF induction on mRNA level, exact composition of calcification induction medium and endpoints to measure calcification will be refined or upgraded. Pathological processes and signaling cascades can be investigated and defined in VICs in the system in detail in ongoing setups. Nevertheless, long term porcine but also human AV tissue cultures investigating arterial blood oxygen concentration vs. hypoxic regulation are envisioned for ongoing research. Therefore, technical MPS development will focus on upscaling the oxygenator to challenge AV tissue culture requirements. Further adaption is necessary to verify the impact of oxygen regulation for dynamic tissue culture settings. This allows the analysis of hypoxia impact dependent e.g. on three layered AV tissue structure, ECM components and cell differentiation.

### Electronic supplementary material

Below is the link to the electronic supplementary material.

Supplementary Material 1

## Data Availability

The datasets used and/or analyses during the current study are available from the corresponding author on reasonable request.
