# Supplementary figures and images for: Oxygenator assisted dynamic microphysiological culture elucidates the impact of hypoxia on valvular interstitial cell calcification
Source: J Biol Eng. 2024 Aug 23;18:45. doi: 10.1186/s13036-024-00441-4 (PMC11342540; doi:10.1186/s13036-024-00441-4)

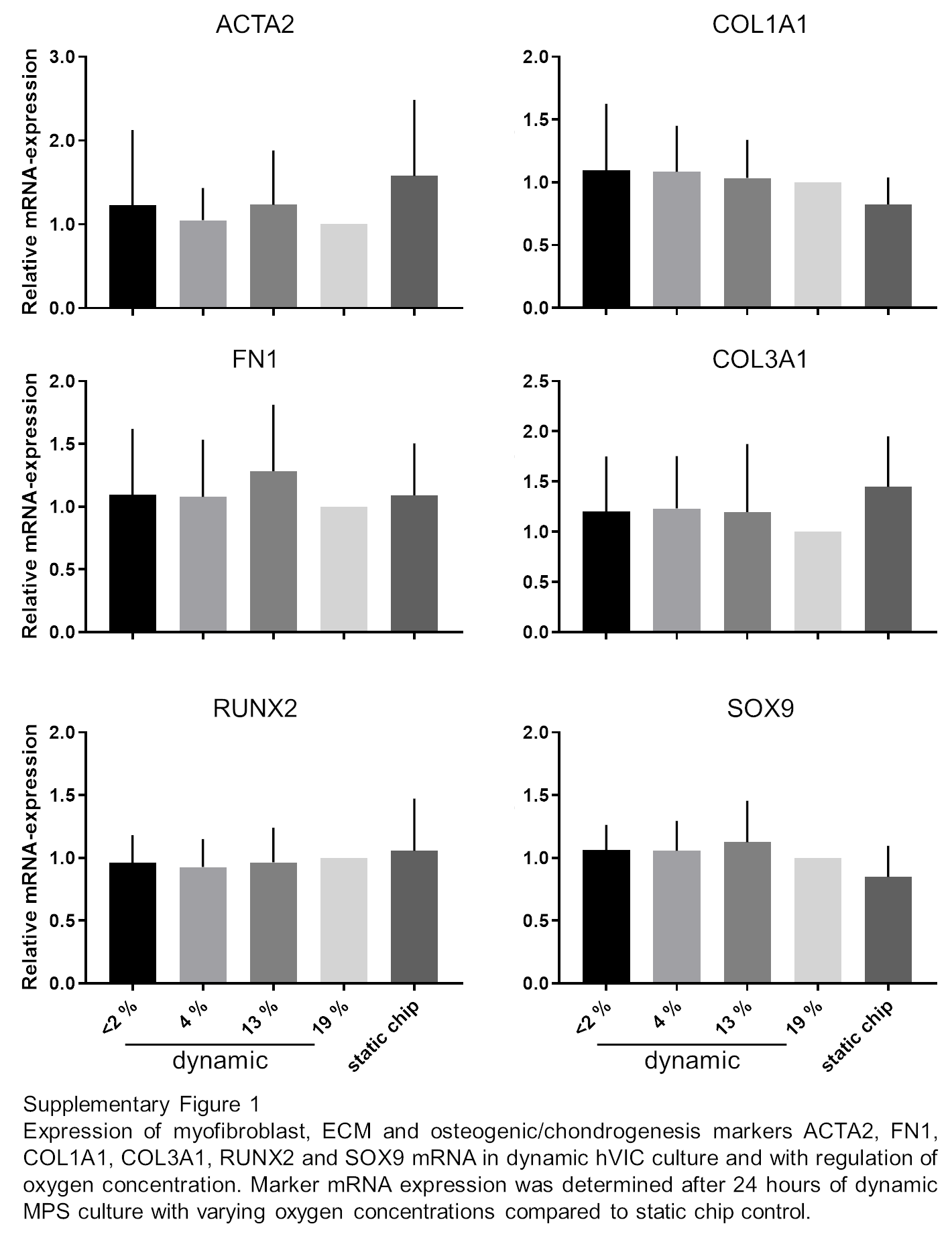

Supplement: Supplementary file 1 — Supplementary Material 1 [file 13036_2024_441_MOESM1_ESM.tif]
